# A Phl p 7-specific IgG4 antibody inhibits allergic patients IgE cross-reactivity to allergens from the EF-hand family: importance of affinity and degree of cross-reactivity

**DOI:** 10.1186/2045-7022-4-S2-O8

**Published:** 2014-03-17

**Authors:** Elisabeth Gadermaier, Louise K James, Mohamed H Shamji, Katrina Blatt, Tetiana Garmatiuk, Rebecca Beavil, Sabine Flicker, Peter Valent, Stephen R Durham, Hannah J Gould, Rudolf Valenta

**Affiliations:** 1 Medical University of Vienna, Center for Pathophysiology, Infectiology and Immunology, Vienna, Austria; 2 King’s College London, Randall Division of Cell and Molecular Biophysics, London, UK; 3 Imperial College London Allergy and Clin Immunol, National Heart/Lung Inst, London, UK; 4 Medical University of Vienna, Vienna General Hospital, Department of Internal Medicine I, Vienna, Austria

## Background

The calcium-binding 2EF-hand protein Phl p 7 from timothy grass pollen is a cross-reactive pollen panallergen that can induce severe clinical symptoms in allergic patients. Recently, a human Phl p 7-specific IgG4 antibody which inhibited IgE binding to Phl p 7 was isolated and characterized.

## Objective

To study epitope-specificity and cross-reactivity of the Phl p 7-specific IgG4 with homologous pollen allergens as well as the extent of cross-protection.

## Methods and results

Sequence comparison showed a comparable sequence identity of approximately 70% between the 2EF-hand pollen allergens (Timothy grass: Phl p 7; Alder: Aln g 4; Birch: Bet v 4; Turnip rape: Bra r 1; Lamb´s quarter: Che a 3; Olive: Ole e 3) and a lower sequence identity of 44% with the 4EF-hand allergen from olive pollen (Ole e 8). While patients´ IgE showed extensive cross-reactivity with the EF-hand allergens, the Phl p 7-specific IgG4 showed limited cross-reactivity. Best cross-reactivity was observed with Ole e 3 whereas the other EF-hand allergens were less recognized by the Phl p 7-specific IgG4 in dot blot experiments. Calcium-depletion experiments showed that the binding of the Phl p 7-specific IgG4 depended on the presence of calcium. Using peptide-specific antisera the binding site of the IgG4 was located in the C-terminal portion of the allergen which contains the second calcium-binding EF-hand motif. Biacore experiments revealed interesting differences regarding the affinity of the Phl p 7-specific IgG4. Phl p 7 and Ole e 3 were recognized with the highest affinity (KD´s: Phl p 7: 2,11x10-9M; Ole e 3: 6,18x10-9M) whereas the affinities to the other cross-reactive allergens was much lower (Bet v 4: 6,26x10-6M; Bra r 1: 6,57x10-6M; Aln g 4: 7,93x10-6M). The cross-inhibition of patients´ IgE reactivity to the EF-hand allergens and the inhibition of allergen-induced basophil activation obtained with the Phl p 7-specific IgG4 followed its intensity of cross-reactivity and affinity with the homologous allergens.

## Conclusion

Our results demonstrate that the Phl p 7-specific IgG4 antibody shows cross-inhibition of allergic patients IgE reactivity and basophil activation induced by homologous allergens according to cross-reactivity and affinity. Funded by grants P23318-B11 and in parts by grants F4607 and F4605 of the Austrian Science Fund (FWF).

